# Neonatal hypoglycaemia and body proportionality in small for gestational age newborns: a retrospective cohort study

**DOI:** 10.1007/s00431-022-04592-8

**Published:** 2022-08-18

**Authors:** Ilke Smits, Liset Hoftiezer, Jeroen van Dillen, Marije Hogeveen

**Affiliations:** 1 Department of Obstetrics and Gynaecology, Radboud University Medical Center, Geert Grooteplein Zuid 10, 6525 GA Nijmegen, The Netherlands; 2 Division of Neonatology, Department of Paediatrics, Amalia Children’s Hospital, Radboud Institute for Health Sciences, Radboud University Medical Center, Geert Grooteplein Zuid 10, 6525 GA Nijmegen, The Netherlands

**Keywords:** Small for gestational age, Symmetric, Asymmetric, Hypoglycaemia, Body proportionality, Newborn

## Abstract

Small for gestational age (SGA) newborns are at risk of developing neonatal hypoglycaemia. SGA newborns comprise a heterogeneous group including both constitutionally small and pathologically growth restricted newborns. The process of fetal growth restriction may result in brain sparing at the expense of the rest of the body, resulting in disproportionally small newborns. The aim of this study was to discover whether body proportionality influences the risk of developing neonatal hypoglycaemia in SGA newborns. A retrospective cohort study was performed in 402 newborns who were SGA without additional risk factors for hypoglycaemia. Body proportionality was classified in two ways: (1) using symmetric (sSGA) or asymmetric (aSGA), defined as head circumference (HC) below or above the 10th percentile, respectively; (2) using cephalization index (HC/birth weight), standardized for gestational age. Hypoglycaemia was observed in 50% of aSGA and 40.9% of sSGA newborns (*P*-value 0.12). Standardized CI in newborns with hypoglycaemia was higher compared to newborns without hypoglycaemia (median 1.27 (1.21–1.35) versus 1.24 (1.20–1.29); (*P* 0.002)). Multivariate logistic regression analyses showed both CI and standardized CI to be associated with the occurrence of hypoglycaemia (OR 1.48 (1.24–1.77) and OR 1.44 (1.13–1.83), respectively). The majority of hypoglycaemic events (96.1%) occurred in the first 6 h after birth.

*   Conclusion*: Body proportionality might be of influence, depending on the classification used. Larger prospective studies with a clear consensus definition of body proportionality are needed.

**Table:** 

**What is Known:** *• Neonatal hypoglycaemia is an important complication in newborns*. *• Small for gestational age (SGA) newborns are more vulnerable to hypoglycaemia*.	
**What is New:** *• Higher incidence of hypoglycaemia was not observed in asymmetric SGA compared to symmetric SGA, but standardized cephalization index was associated with increased likelihood of hypoglycaemia*. *• Consensus-based definitions of body proportionality in newborns are needed*.

**Supplementary Information:**

The online version contains supplementary material available at 10.1007/s00431-022-04592-8.

## Introduction

### Background

Neonatal hypoglycaemia is an important complication in newborns [1], especially in those with fetal growth restriction [1–3]. The concept of fetal growth restriction (FGR) refers to a pathological process resulting in a fetus who does not reach its full growth potential during pregnancy [2]. Because FGR is difficult—if not impossible—to diagnose with certainty, many different proxy measures exist to denote whether a newborn may have suffered FGR. After birth, the classification “small for gestational age” (SGA) is often used as a proxy for FGR. SGA is a statistical definition referring to a birthweight below a predefined threshold, generally the 10th percentile (p10) for gestational age. The group of SGA newborns comprises both pathologically growth-restricted newborns and genetically small but otherwise healthy newborns [3].

SGA newborns may be proportionately (i.e., symmetric) or disproportionately (i.e., asymmetric) small. In symmetric SGA (sSGA) newborns, anthropometric measurements are equally affected, resulting in a normal brain-to-body ratio. They may be either constitutionally small or may have suffered early intrauterine infection (e.g., CMV) or a genetic disorder (e.g., chromosomal abnormalities) [4]. Asymmetric SGA (aSGA) newborns have an increased brain-to-body ratio; this type of growth restriction is generally attributed to placental insufficiency due to maladaptation (often in combination with maternal hypertension), multiple pregnancy, maternal malnutrition, or maternal smoking [5]. Placental insufficiency leads to brain sparing when most of the energy is used for brain development to the detriment of the rest of the body [5, 6]. These aSGA newborns may be especially vulnerable to hypoglycaemia due to lower glycogen stores, higher energy requirements, decreased gluconeogenesis, decreased counter regulatory hormones, fewer alternative fuel sources (such as ketones and free fatty acids), and increased insulin sensitivity [7].

In a retrospective study from India involving 127 SGA newborns, Bhat et al. observed a higher incidence of neonatal hypoglycaemia in asymmetric compared with symmetric SGA newborns (25 versus 20%, respectively) [8]. In a prospective study from Spain, Nieto also found a higher incidence of neonatal hypoglycaemia in asymmetrical SGA compared to symmetric SGA in 185 term newborns (25.4 versus 3.9% respectively, *P*-value < 0.01) [9]. Both authors used the ponderal index (i.e., weight/length^3^) to classify body proportionality, where a low ponderal index (PI) indicates disproportionate or asymmetric growth restriction. However, the PI assesses whether length is spared at the expense of weight and not whether head growth is spared at the expense of the remaining body.

### Objective

In our hospital, all SGA newborns are routinely screened for hypoglycaemia [10]. If the risk of hypoglycaemia is indeed affected by body proportionality, risk stratification might be improved and may result in fewer but more targeted blood samplings. Therefore, our aim was to assess the association between body proportionality and the risk of hypoglycaemia in SGA newborns.

## Methods

### Study design

We performed a retrospective cohort study, using data from newborns who were considered at risk of neonatal hypoglycaemia according to local protocol (prematurity, large for gestational age, SGA, maternal pre-existent, and gestational diabetes).

### Setting

The data was collected from a 4-year period (01-01-2010 until 01-01-2014) at the Radboudumc Amalia Children’s Hospital, Nijmegen, the Netherlands [11]. At that time, SGA was defined as birthweight < p10 based on the population-based birthweight standard by Visser et al. [12].

### Participants

Our study population was limited to term SGA newborns of whom plasma glucose levels during the first 24 h were available. In order to study predominantly the effect of SGA, newborns who met the following criteria were excluded: death within the first 24 h after birth, severe asphyxia (i.e., Apgar-score 5 min postpartum ≤ 3), gestational age (GA) < 37 weeks, maternal use of Labetalol during pregnancy, and the presence of any type of diabetes [13, 14].

### Variables

Neonatal characteristics included HC, BW, and gestational age at delivery. Additionally, maternal characteristics such as age, presence of FGR, and hypertension were recorded.

Body proportionality was classified in two ways: (1) using symmetric (sSGA) or asymmetric (aSGA), defined as head circumference (HC) below or above the 10th percentile, respectively; (2) using cephalization index (HC/birth weight), standardized for gestational age.

There is no internationally agreed definition of body proportionality in SGA newborns. Besides the PI which does not directly indicate brain sparing, several methods to determine brain-to-body ratio have been previously described to classify body proportionality, although not in relation to the incidence of hypoglycaemia [4, 15–19]. HC is a simple measurement which is often measured routinely before discharge. As for birthweight, reference values are available to assess normality relative to gestational age. Unfortunately, both the former and current Dutch standards do not contain a matching HC curve [12, 20]. Therefore, eligible newborns were reclassified as SGA or not using the birthweight standards of Niklasson, which closely resemble the current Dutch birthweight charts and have a matching HC curve [21]. If the neonatal HC percentile was < p10, the newborn was classified as symmetric SGA (sSGA). Conversely, if the neonatal HC was ≥ p10, the newborn was classified as asymmetric SGA (aSGA) [10].

The cephalization index (CI) is a ratio of head circumference to birthweight [22]. We calculated the CI (i.e., (HC in cm)/(birthweight in g)) for each newborn and compared the values between newborns with and without hypoglycaemia. To account for the effect of GA, we also calculated a “standardized CI” for each individual by dividing each individual CI by a “mean CI for GA.” These “means” were calculated by dividing the gender- and GA-specific means of HC and birthweight by Niklasson and Albertsson-Wikland [21].

Our primary outcome was the occurrence of neonatal hypoglycaemia defined in our local protocol as a plasma glucose concentration lower than 2.2 mM in the first 24 h after birth. Our secondary outcome was the timing of the first hypoglycaemia in hours after birth.

### Data sources

The variables were all extracted from the patient files. Plasma glucose concentrations were measured routinely 1, 3, 6, 12, and 24 h after birth, using a point-of-care (POC) glucose meters such as HemoCue Glu201DM, Nova StatStrip GluCard memory PC, and Roche OMNI-56 blood gas. In case of hypoglycaemia, capillary blood was sent to the laboratory for confirmation.

### Bias

Important risk factors for hypoglycaemia are gestational age (prematurity and postmaturity), maternal diabetes, low birth weight (< 2500 g), and twin delivery [23, 24]. Maternal diabetes of any type and GA < 37 weeks were exclusion criteria. The other potential confounders, namely postmaturity, low birth weight (< 2500 g), and twin delivery, were analyzed and adjusted for if indicated (see “Results” section).

### Statistical methods

Descriptive statistics were used to study demographic characteristics, including proportions and mean (SD). The Chi-square or Fisher exact tests were used for categorical variables and the Students *T* or Mann–Whitney *U* tests were used for continuous variables (dependent on variable distributions). The potential association between hypoglycaemia and relevant variables was evaluated using univariate logistic regression analyses. Variables with *P*-value < 0.20 were considered for multivariate analysis. Stepwise backward multivariate analyses were performed and odds ratios and their 95% CI and *P*-values were calculated. For multiple regression analysis, two models were used: model 1 with body proportionality expressed as aSGA or sSGA; model 2 with body proportionality expressed as standardized CI. Collinearity was checked using Pearson correlation coefficients, tolerance, and variance inflation factor (VIF). Goodness of fit was assessed by the Hosmer and Lemeshow test. A receiver operating characteristic (ROC) curve was constructed to evaluate the performance of the standardized CI as an instrument to predict hypoglycaemia. The timing of the first hypoglycaemia was analyzed using the log rank test and shown in the Kaplan–Meier curve. Results were considered significant if the *P*-value was < 0.05. Data was analyzed using IBM SPSS Statistics for Windows, version 25 (IBM Corporation Inc., Armond, NY, USA).

This study was exempt from Regional Ethics Review Board approval, under the legal requirements for clinical research in the Netherlands (case number 2020-6531).

## Results

### Participants

**Fig. 1:**
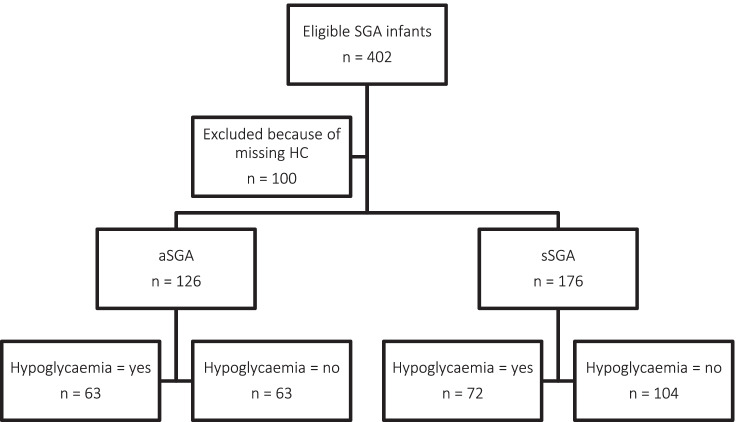
Flow diagram of study participants

In this study, 402 SGA newborns were eligible; 100 newborns (24.9%) could not be categorized due to missing HC and were excluded from further analysis. Of the 302 included SGA newborns, 126 were aSGA (41.7%) and 176 were sSGA (58.3%) (Fig. 1).

### Descriptive data

Maternal and neonatal characteristics are shown in Table 1. FGR was suspected in only 21.2% of the included cases (Table 1). The demographics in Table 1 showed significant differences between the aSGA and sSGA groups on birthweight *Z*-scores and percentiles, HC indices, and gender.

**Table 1:** Baseline demographic characteristics of the study population

	**Study population** **(*n* = 302)**	**aSGA** **(*n* = 126)**	**sSGA** **(*n* = 176)**	***P* value**^**b**^
Female gender (*n* (%))	162 (53.6)	78 (61.9)	84 (47.7)	0.015^*^
GA, weeks (mean (SD))	39.4 (1.3)	39.3 (1.3)	39.4 (1.3)	0.712
BW, grams (mean (SD))	2623 (308)	2642 (301)	2608 (314)	0.344
BW *Z*-score (mean (SD))	−2.3 (0.6)	−2.2 (0.6)	−2.4 (0.6)	0.006^*^
BW percentile (mean (SD))	1.9 (1.9)	2.3 (2.1)	1.6 (1.7)	0.002^*^
LBW (< 2500 g) (*n* (%))	106 (35.1)	40 (31.7)	66 (37.55)	0.302
HC, cm (mean (SD))	33.2 (1.2)	34.1 (1.1)	32.7 (1.0)	< 0.001^*^
HC *Z*-score (mean (SD))	−1.4 (0.8)	−0.7 (0.6)	−1.9 (0.5)	< 0.001^*^
HC percentile (mean (SD))	13.5 (16.3)	26.5 (18.3)	4.2 (2.8)	< 0.001^*^
Cephalization index (median (IQR))	0.013 (0.012–0.014)	0.013 (0.012–0.014)	0.012 (0.012–0.014)	0.014^*^
Standardized CI (median (IQR))	1.25 (1.20–1.31)	1.27 (1.22–1.32)	1.24 (1.19–1.30)	0.004^*^
Singleton pregnancy (*n* (%))	274 (90.7)	111 (88.1)	163 (92.6)	0.182
Maternal hypertension (*n* (%))	56 (18.5)	20(15.9)	36 (20.5)	0.312
Maternal smoking (*n* (%))	64 (21.2)	24 (19.0)	40 (22.7)	0.440
Maternal BMI (mean (SD))	23.8 (4.6)	24.4 (5.4)	23.4 (4.0)	0.111
Maternal BMI > 30 (*n* (%))	23 (7.6)	14 (11.1)	9 (5.1)	0.053
Maternal age (mean (SD))	31.1 (5.1)	31.4 (4.8)	30.9 (5.3)	0.335
Maternal age > 35 years (*n* (%))	62 (20.5)	27 (21.4)	35 (19.9)	0.744
Antenatal suspicion of FGR (*n* (%))^a^	64 (21.2)	29 (23.0)	35 (19.9)	0.512

*aSGA* asymmetric small for gestational age, *sSGA* symmetric small for gestational age, *GA* gestational age, *BW* birthweight, *LBW* low birthweight, *HC* head circumference, *CI* cephalization index, *BMI* body mass index, *FGR* fetal growth restriction

^*^P value < 0.05 is considered statistically significant

^a^FGR was defined according to the Dutch guideline, i.e., estimated fetal weight (EFW) and/or abdominal circumference (AC) < p10 and/or a decrease of > 20 percentiles during serial ultrasound measurements with at least a two week interval [28]

^b^Frequencies were compared using Chi-square test; means were compared using Student’s *T*-test

### Main results

The overall incidence of hypoglycaemia was 44.7%. Of the aSGA newborns, 50.0% experienced neonatal hypoglycaemia, compared to 40.9% of the sSGA newborns (*P*-value 0.12) (Fig. 1). CI decreased with increasing GA (Supplement 1a). All newborns had standardized CIs above 1.0 (Supplement 1b). The standardized CI was slightly higher in newborns who experienced hypoglycaemia versus those who did not experience hypoglycaemia (median 1.27 (IQR 1.21–1.35) versus 1.24 (IQR 1.20–1.29); *P*-value 0.002). Although very small due its scale, the difference in CI between newborns with versus without hypoglycaemia was also significant (median CI 0.12 (0.12–0.13) versus median CI 0.13 (0.12–0.14); *P* value < 0.001).

ROC analysis (Supplement 2) showed an area under the curve (AUC) of 0.602 (95% CI 0.538–0.667). Univariate analyses are presented in Table 2. Multicollinearity diagnostics showed high correlation between birthweight *Z*-score and (standardized) CI and between GA and CI (Supplement 3), which were therefore excluded from further analyses. Although correlation between body proportionality and (standardized) CI was low (Supplement 3), we chose to create separate models for each predictor. To facilitate interpretation of the results, CI and standardized CI were linearly transformed by multiplying the original values by 10^3 and 10^1, respectively. Multiple pregnancy was univariately associated with hypoglycaemia (at *P* < 0.20) but not in the multivariate analyses. Body proportionality (i.e., aSGA versus sSGA) was not associated with hypoglycaemia, taking into account GA and gender (Table 3). Both CI and standardized CI, however, were significantly associated with increased odds of hypoglycaemia (Table 3). The Hosmer and Lemeshow statistic indicated a good fit for all models (*P* > 0.05; results not shown).

**Table 2:** Univariate logistic regression analyses with occurrence of hypoglycaemia as outcome

Variable (reference category)	*P*-value	OR	95% confidence interval
***Maternal factors:***			
BMI (continuous)	0.622	0.99	0.93; 1.04
BMI > 30 (no.)	0.902	0.95	0.40; 2.23
Maternal age (continuous)	0.925	1.00	0.95; 1.04
Maternal age > 35 years (no.)	0.288	0.73	0.42; 1.30
Smoking (no.)	0.694	1.12	0.64; 1.94
Hypertension (no.)	0.367	0.76	0.42; 1.38
***Newborn factors:***			
Gender (female)	0.051	1.58	1.00–2.49
Multiple pregnancy (no.)	0.169	1.74	0.79; 3.81
**GA**	**0.001**	**0.96**	**0.94; 0.98**
**BW *Z*-score**	**0.011**	**0.61**	**0.41; 0.89**
Body proportionality (sSGA)	0.118	1.44	0.91; 2.29
CI*10^3	**< 0.001**	**1.43**	**1.20; 1.70**
Standardized CI*10^1	**0.001**	**1.49**	**1.17; 1.89**

*OR* odds ratio, *BMI* body mass index, *GA* gestational age, *BW* birthweight, *sSGA* symmetric small for gestational age, *CI* cephalization index

**Table 3:** Multivariate regression analyses with occurrence of hypoglycaemia as outcome

		*P*-value	OR	95% confidence interval	
				Lower	Upper
Model 1^a^	Body proportionality (reference = sSGA)	0.068	1.56	0.97	2.53
	Gestational age (days)	0.001	0.96	0.93	0.98
	Gender (reference = female)	0.027	1.71	1.06	2.75
	Constant	0.002	59996		
Model 2^b^	Standardized CI*10^1	0.003	1.44	1.13	1.83
	Gender (reference = female)	0.036	1.66	1.03	2.68
	Gestational age (days)	0.005	0.96	0.94	0.99
	Constant	0.216	170.77		
Model 3^c^	CI*10^3	< 0.001	1.48	1.24	1.77
	Gender (reference = female)	0.012	1.85	1.14	2.99
	Constant	< 0.001	0.004		

*OR* odds ratio, *sSGA* symmetric SGA, *CI* cephalization index

^a^Body proportionality expressed as aSGA or sSGA

^b^Body proportionality expressed as standardized CI

^c^Body proportionality expressed as CI

### Other results

The majority of the SGA newborns (96.1%) developed hypoglycaemia within the first 6 h; the remaining 5 SGA newborns developed their first hypoglycaemia at 24 h after birth (3.9%) (Supplement 4). No significant difference in timing of hypoglycaemic events between sSGA and aSGA newborns was found (*P*-value 0.15), although 4 out of 5 newborns who developed hypoglycaemia at 24 h were sSGA (80%).

## Discussion

### Key results

Overall, 44.7% of SGA newborns developed hypoglycaemia. We observed a higher although not statistically different incidence of hypoglycaemia in aSGA (50.0%) compared to sSGA (40.9%) newborns. However, both CI and standardized CI were significantly associated with increased odds of hypoglycaemia. These results suggest that body proportionality may affect the risk of hypoglycaemia in SGA newborns, depending on the definitions.

### Limitations

Our study has several limitations. First of all, the lack of a Dutch newborn HC compelled us to use an alternative birthweight standard [21]. Although the Swedish birthweight charts are quite similar to the current Dutch birthweight charts, the 10th percentiles for both male and female newborns exceed the cut-off values for SGA that were applied during the study period [21]. This means that at the time, more newborns would have been classified as SGA—and subjected to glucose controls—if the Swedish charts had been used for risk stratification. Because the newborns who were “missed” would have had slightly higher birthweights, we expect an overall lower incidence of hypoglycaemia. Whether this would affect our results remains a question. Another limitation inherent to our retrospective design is the amount of missing data. Unfortunately, 24.9% of the HC measurements were missing in newborns who fulfilled our inclusion criteria. The incidence of hypoglycaemia in the excluded newborns was 40.0% (results not shown).

### Interpretation

Two others studies (Bhat et al. and Nieto et al.) found a higher incidence of hypoglycaemia in aSGA compared to sSGA newborns [8, 9]. However, there were large differences in study methodology which complicates the comparison between the separate studies. Both Bhat et al. and Nieto et al. used the PI to classify body proportionality. Unfortunately, length at birth is not routinely registered in our hospital; in only 21% of our cases, the measurement was available and the accuracy of these measurements may be questionable [25]. To the best of our knowledge, it is unknown whether length and head circumference are preserved to the same extent in fetuses who experience FGR. We were unable to identify studies that investigated the association between the PI and HC-relative-to-birthweight. An analysis of 182 stillborn autopsies showed a poor correlation between the PI and brain/liver ratio [5], thus suggesting poor correlation with brain sparing.

In our study, we found a much higher overall incidence of hypoglycaemia (44.7%) in comparison to the studies of Bhat et al. (25.2%) and Nieto et al. (10.8%). This variability in incidences may be partly due to different definitions used for the classification of SGA, different in- and exclusion criteria for the study population, and different approaches to the detection and prevention of hypoglycaemia. For example, Bhat et al. defined SGA as a birthweight below 1 SD (which is approximately equivalent to p16), whereas in our study, newborns were classified as SGA when birthweight was < p10 [8]. Since there is an association between the severity of SGA and the risk of hypoglycaemia, the inclusion of relatively bigger neonates by Bhat et al. might explain the lower overall incidence of hypoglycaemia [26]. Interestingly though, mean birthweight in term SGA infants was 2120 ± 235 compared to 2622 ± 308 g in our population. This illustrates the large differences in the distribution of birthweight between the populations and complicates comparison of results. An even lower incidence was found by Nieto et al. where only 10.8% of all SGA newborns developed hypoglycaemia. Although their definition of SGA was similar (i.e., birthweight < p10), their cut-off level for hypoglycaemia was < 35 mg/dL (i.e., < 1.9 mM) compared to < 2.2 mM in our study [9]. Nieto et al. also described a relatively larger difference between the separate groups, i.e., a 6.5 times higher risk in aSGA versus sSGA newborns, compared to a relative risk increase of 1.2 in our study (ns) and 1.3 in the study of Bhat et al. [8, 9]. This suggests that aSGA newborns may be at risk of more severe hypoglycaemia compared to sSGA newborns. When we repeated our analyses using the cut-off proposed by Nieto et al. we found an overall incidence of 25.2%, 32.8% in aSGA newborns, and 21.2% in sSGA newborns (*P*-value 0.024). Although this is a statistically significant difference, the relative risk in aSGA newborns is still only 1.5 compared to sSGA newborns and the overall incidence remains high (i.e., 25.2 versus 10.8% in the study by Nieto et al.). Information on symptomatology would have been helpful to determine the clinical relevance of our findings.

We defined aSGA as the HC being “preserved” or above the statistical limit for gestational age, using, similar to our SGA cut-off for birthweight, the 10th percentile as a cut-off [21]. Although convenient for classification and easily understandable, dichotomization causes oversimplification which results in a loss of information [1]. For example, a newborn with a birthweight at p9.9 and a HC at p10.1 is classified as asymmetric, whereas a newborn with a birthweight at p1.0 and a HC at p9.9 is classified as symmetric. Clearly, the latter has more evidence of relative brain sparing. Theoretically, a continuous measure such as the CI could resolve this issue. Moreover, it could be used in newborns who experienced FGR but are not SGA at birth and could be used in populations for which no population-specific birthweight or HC references exist. The CI was previously described in relation to neurodevelopment, where higher brain-to-body ratio reflected a greater degree of brain vulnerability [22]. Harel et al. constructed a normal curve and extrapolated a CI for each newborn, using term infants as a reference [22]. We hypothesized that higher brain-to-body ratio might also be associated with an increased risk of hypoglycaemia. The standardized CIs were all > 1.0, suggesting an inversely proportional relation between birthweight and brain-to-ratio, where newborns with lower birthweights tend to have relatively larger HCs. This might also explain why the (standardized) CIs were only slightly lower in sSGA compared to aSGA newborns, the former group being relatively smaller (i.e., lower birthweight *Z*-scores and percentiles). Our results showed a slightly higher (median) CI and standardized CI in newborns with hypoglycaemia, but the AUC was low indicating poor discriminative ability. Multivariate logistic regression analyses showed that higher (standardized) CI was associated with a significantly higher risk of hypoglycaemia. Newborns who experienced FGR but were not SGA at birth are not routinely subjected to glucose controls, making it challenging to determine whether an increased brain-to-body ratio irrespective of birthweight percentile could be associated with an increased risk of hypoglycaemia.

### Generalizability

The aim of our study was to assess the association between body proportionality and risk of hypoglycaemia in SGA newborns. A clinically relevant difference in the incidence of hypoglycaemia could justify a more targeted approach in glucose controls. As stated previously, not all SGA newborns have experienced FGR and vice versa. Others have suggested alternative strategies to distinguish between newborns who are constitutionally small versus those pathologically growth-restricted and at risk of adverse outcomes. A recent study by Beune et al. suggested a consensus definition on “growth restriction in the newborn,” aiming to detect pathologically small newborns [27]. Among other items, the “presence of maternal risk factors” was included in the definition, although not further specified. In our study, the presence of hypertensive disorders and other maternal characteristics did not influence the risk of hypoglycaemia. At this point in time, without consensus definitions to classify SGA and body proportions, it is difficult to extrapolate our results to other populations.

### Conclusion

The association between body proportionality and neonatal hypoglycaemia in SGA newborns needs further evaluation using consensus-based definitions of growth restriction and body proportionality. The (standardized) CI may be a promising tool. HC and length at birth should be routinely measured and registered in the Dutch perinatal database to enable the future development of Dutch HC and length at birth reference values. Ideally, our hypothesis should be evaluated in a prospective study where besides anthropometric measurements also clinical symptoms are collected.

## Supplementary Information

Below is the link to the electronic supplementary material.

Supplementary file1: Cephalization index (i.e., individual brain-to-body ratio of each newborn) in relation to gestational age (GA) (EPS 1430 KB)

Supplementary file2: Standardized cephalization index (i.e., individual brain-to-body divided by mean brain-to-body ratio) in relation to gestational age (GA) (EPS 1427 KB)

Supplementary file3: Area under the curve of standardized CI and risk of hypoglycaemia (EPS 1326 KB)

Supplementary file4: Pearson correlationcoefficients and collinearity statistics (DOCX 19 KB)

Supplementary file5: Kaplan Meier curve fortiming of first hypoglycaemia for sSGA and aSGA newborns (EPS 1411 KB)

Supplementary file6: STROBE Statement. Checklist of items that should be included in reports of ***cohort studies*** (DOCX 23 KB)

## Data Availability

Data was extracted from the electronic patient files.

## Abbreviations

aSGA: Asymmetric small for gestational age
CI: Cephalization index
FGR: Fetal growth restriction
GA: Gestational age
HC: Head circumference
PI: Ponderal index
ROC: Receiver operating characteristic
SGA: Small for gestational age
sSGA: Symmetric small for gestational age

## Code availability

SPSS.
