# Supplementary material for: Neonatal hypoglycaemia and body proportionality in small for gestational age newborns: a retrospective cohort study
Source: Eur J Pediatr. 2022 Aug 18;181(10):3655–62. doi: 10.1007/s00431-022-04592-8 (PMC9508048; doi:10.1007/s00431-022-04592-8)
Supplement: Supplementary file 4 — Supplementary file4: Pearson correlationcoefficients and collinearity statistics (DOCX 19 KB) [file 431_2022_4592_MOESM4_ESM.docx]

Supplement 3 Pearson correlation coefficients and collinearity statistics.

| **Pearson correlation coefficients** | | | | | | | |
| --- | --- | --- | --- | --- | --- | --- | --- |
|  | Gender | Gestational age (days) | Birthweight Z-score | Body proportionality | Multiple pregnancy | Cephalization index | Standardized cephalization index |
| Gender | **1.000** | 0.000 | -0.034 | -0.140 | 0.000 | -0.111 | -0.029 |
| Gestational age (days) | 0.000 | **1.000** | 0.091 | -0.021 | -0.270 | **-0.652** | -0.153 |
| Birthweight Z-score | -0.034 | 0.091 | **1.000** | 0.157 | -0.058 | **-0.740** | **-0.919** |
| Body proportionality | -0.140 | -0.021 | 0.157 | **1.000** | 0.077 | 0.125 | 0.132 |
| Multiple pregnancy | 0.000 | -0.270 | -0.058 | 0.077 | **1.000** | 0.220 | 0.098 |
| Cephalization index | -0.111 | **-0.6521** | **-0.740** | 0.125 | 0.220 | **1.000** | **0.841** |
| Standardized cephalization index | -0.029 | -0.153 | **-0.919** | 0.132 | 0.098 | **0.841** | **1.000** |

| **Collinearity statistics CI** | | |
| --- | --- | --- |
|  | Tolerance | VIF |
| Gender | 0.799 | 1.252 |
| Gestational age (days) | 0.124 | 8.038 |
| Birthweight Z-score | **0.084** | **11.858** |
| Body proportionality | 0.507 | 1.971 |
| Multiple pregnancy | 0.920 | 1.087 |
| Cephalization index | **0.048** | **20.693** |

| **Collinearity statistics sCI** | | |
| --- | --- | --- |
|  | Tolerance | VIF |
| Gender | .974 | 1.027 |
| Gestational age (days) | .883 | 1.132 |
| Birthweight Z-score | **.073** | **13.668** |
| Body proportionality | .472 | 2.117 |
| Multiple pregnancy | .920 | 1.087 |
| Standardized cephalization index | **.073** | **13.763** |
